# Role of the dengue vaccine TAK-003 in an outbreak response: Modeling the Sri Lanka experience

**DOI:** 10.1371/journal.pntd.0012376

**Published:** 2024-08-22

**Authors:** LakKumar Fernando, Randee Kastner, Pujitha Wickramasinghe, Asvini D. Fernando, Dulanie Gunasekera, Van Hung Nguyen, Mengya Liu, Inge LeFevre, Derek Wallace, Nicolas Folschweiller, Shibadas Biswal

**Affiliations:** 1 Centre for Clinical Management of Dengue & Dengue Haemorrhagic Fever, Negombo General Hospital, Negombo, Sri Lanka; 2 Takeda Vaccines, Inc., Boston, Massachusetts, United States of America; 3 University of Colombo, Colombo, Sri Lanka; 4 Faculty of Medicine, University of Kelaniya, Kelaniya, Sri Lanka; 5 Faculty of Medical Sciences, University of Sri Jayawardenenpura, Colombo, Sri Lanka; 6 VHN Consulting, Montreal, Quebec, Canada; 7 Takeda Pharmaceuticals International AG, Zurich, Switzerland; University of Pittsburgh, UNITED STATES OF AMERICA

## Abstract

**Background:**

Outbreaks of dengue can overburden hospital systems, drastically reducing capacity for other care. The 2017 dengue serotype 2 (DENV-2) outbreak in Sri Lanka coincided with vaccination in an ongoing phase 3 efficacy trial of a tetravalent dengue vaccine, TAK-003 (NCT02747927). Here, we present data on the efficacy of TAK-003 following two doses of the vaccine administered 3 months apart in participants aged 4–16 years in Sri Lanka. In addition, we have used the 2017 outbreak dynamics to model the potential impact of TAK-003 on virologically confirmed dengue (VCD) cases and hospitalizations during an outbreak situation.

**Methodology/principal findings:**

Modeling was performed using an age-structured, host-vector, spatial and stochastic transmission model, assuming 65% vaccine coverage and 30 days until initiation of vaccination. Efficacy of TAK-003 against VCD and hospitalized VCD cases was based on data against DENV-2 from the first year of the phase 3 trial. Vaccine efficacy and safety findings in Sri Lanka were in line with those of the overall trial population. The efficacy estimates in Sri Lanka up to the first 12 months after the second dose of TAK-003 were 94.7% and 95.7% against VCD and hospitalized VCD cases, respectively. Modeling of the trial data over an extended geographic area showed a substantial reduction in cases and a flattening of outbreak curves from TAK-003 use. The baseline vaccination scenario (initiation at 30 days, 65% target coverage, vaccine effective at 14 days, 70% hospitalization rate, VE of 95% for VCD and 97% for hospitalized VCD, and 47% for asymptomatic) resulted in a 69.1% reduction in VCD cases and 72.7% reduction in VCD hospitalizations compared with no vaccination. An extreme high scenario (vaccination initiated at Day 15, 80% coverage rate, baseline VE) resulted in 80.3% and 82.3% reduction in VCD and VCD hospitalizations, respectively. Vaccine performance, speed of vaccination campaign initiation, and vaccine coverage were key drivers in reducing VCD cases and hospitalizations.

**Conclusions/significance:**

Overall, the study and modeling results indicate that TAK-003 has the potential of meaningful utility in dengue outbreaks in endemic areas.

## Author summary

Dengue is a viral disease transmitted to humans by infected mosquitoes. Outbreaks of dengue occur in many tropical and subtropical countries and can place a big strain on health care services. In Sri Lanka, dengue occurs throughout the year, with outbreaks every few years. In 2017, Sri Lanka experienced a large dengue outbreak caused by dengue virus serotype 2. By chance, a clinical trial of a dengue vaccine (TAK-003) was taking place at the same time, which allowed us to look at how well the vaccine could work to control an outbreak. Because all trial sites were in a small region of Sri Lanka, we used a model to look at the vaccine’s potential impact over a wider area. Data from the clinical trial showed that the vaccine lowered the risk of symptomatic dengue infection and the risk of hospitalization owing to dengue. In addition, using epidemiological data from Sri Lanka at the time of the outbreak, the model showed that vaccination would be linked to a reduction in dengue cases and related hospitalizations compared with no vaccination, and would “flatten the curve” of the outbreak. Overall, our study shows that TAK-003 could be useful in controlling a dengue outbreak.

## Introduction

Outbreaks of infectious diseases can substantially overburden health care systems; therefore, flattening the peak of cases and hospitalizations is a recognized approach to manage outbreaks [1,2]. Dengue, an arboviral infection transmitted predominantly by *Aedes aegypti* mosquitoes, occurs throughout the year in Sri Lanka, with seasonal epidemics occurring every few years, mostly at times of increased rainfall [1]. Clinical management of dengue cases in Sri Lanka involves multiple evaluations, such as monitoring platelet counts and ultrasonography, which results in a high rate of hospitalization of dengue cases [2]. Therefore, a dengue outbreak can quickly lead to a high level of hospital utilization and can drastically reduce capacity for other hospital care.

In 2017, Sri Lanka experienced its largest dengue outbreak to date, resulting in 186,101 reported cases and 440 deaths [1]. In July 2017 alone, 41,121 cases were recorded [3]. Most cases were among teenagers and young adults, with the highest dengue incidence rate occurring in persons aged 20–29 years [1]. Whilst the overall incidence rate was reported as 866 cases per 100,000 population, the incidence rate was highest in Colombo (1419 per 100,000 population) and Gampaha (1323 per 100,000 population) [1]. Dengue virus serotype 2 (DENV-2) was identified as the predominant serotype during the outbreak [1,4]. By chance, the country’s 2017 dengue outbreak coincided with an ongoing phase 3, randomized, clinical trial assessing the efficacy of two doses of Takeda’s TAK-003 administered 3 months apart. TAK-003 is a live-attenuated tetravalent dengue vaccine based on a DENV-2 virus backbone (TDV-2) with three chimeric viruses containing the DENV-1, -3, and -4 components [5]. TAK-003 demonstrated long-term efficacy and safety in dengue prevention, with vaccine efficacy (VE) against all four serotypes in baseline seropositive participants and against DENV-1 and DENV-2 in baseline seronegative participants. Efficacy was not shown against DENV-3 and was inconclusive against DENV-4 in baseline seronegative participants (S1 Table) [6]. The TIDES trial included four sites in the Colombo and Gampaha districts. Participants had received trial vaccinations before the peak of the outbreak, with administration of the first dose between November 2016 and March 2017 and the second dose between February and July 2017. The Sri Lankan trial data, therefore, provide an opportunity to gain insight into the potential impact that TAK-003 can have during a DENV-2 outbreak.

Here, we provide an exploratory and descriptive analysis of the efficacy of TAK-003 in participants taking part in the phase 3 trial who were enrolled at sites in Sri Lanka. In addition, we use results from an age-structured, high spatial resolution, stochastic transmission model to evaluate the potential impact of TAK-003 vaccination on cases of virologically confirmed dengue (VCD) and VCD cases leading to hospitalization in an outbreak situation based on the VE profile against the DENV-2 serotype and the epidemiology of the 2017 outbreak.

## Methods

### Ethics statement

The trial protocol and associated documents were approved by the relevant authorities and ethics committees prior to initiation of the trial, as per local regulations (S2 Table). Written informed assent/consent was obtained from all participants or their parents/legal guardians before enrolment as applicable. The trial is registered at ClinicalTrials.gov (NCT02747927).

### Trial design and participants

Full details of the trial design have been published previously [2,7–9]. In brief, the phase 3, double-blind, randomized, placebo-controlled trial enrolled healthy children aged 4–16 years living in eight dengue-endemic countries in Asia and Latin America. Participants were randomized 2:1 to receive two doses of TAK-003 or placebo 3 months apart.

**Fig 1:**
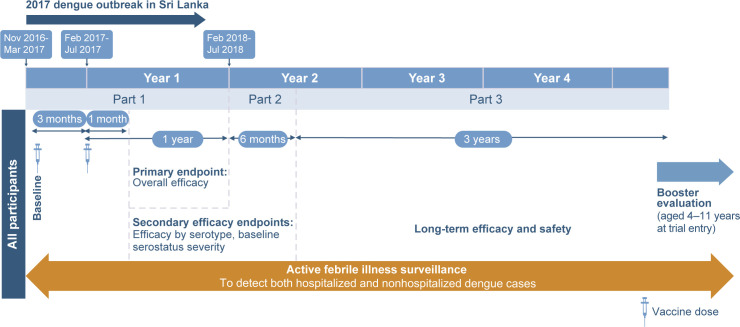
Design of the phase 3 randomized trial.

As shown in Fig 1, the ongoing trial includes active surveillance during parts 1 and 2 and modified active surveillance in part 3 (see supplementary appendix in Biswal *et al* 2019 [7] for details), as well as in the booster evaluation phase. Throughout the trial, participants or their parents/guardians were contacted at least once a week to encourage reporting of febrile illness (defined as fever of ≥ 38°C on any 2 of 3 consecutive days) to allow identification of all symptomatic dengue cases. Cases were confirmed via a serotype-specific reverse transcription polymerase chain reaction, i.e., VCD in the acute samples (full details have been reported previously [7]). Hospitalization of cases was per the local standard of care [10]. The Dengue Case Severity Adjudication Committee independently assessed all hospitalized VCD cases via blinded review using predefined criteria to determine severe dengue [7]. Separately, all VCD cases were assessed for dengue hemorrhagic fever using a programmed algorithm to analyze data based on 1997 World Health Organization criteria for dengue hemorrhagic fever [11].

In this manuscript, safety and efficacy data are presented for the safety set participants in Sri Lanka (i.e., trial participants who received at least one dose of TAK-003 or placebo). Data are presented for the first 15 months of the trial because this period is relevant to an outbreak scenario, as well as from the latest available time point (approximately 57 months from the start of the trial). Additional information on the safety and efficacy methodology and results can be found in S1 Text.

### Modeling the impact of vaccination on outbreak management

**Fig 2:**
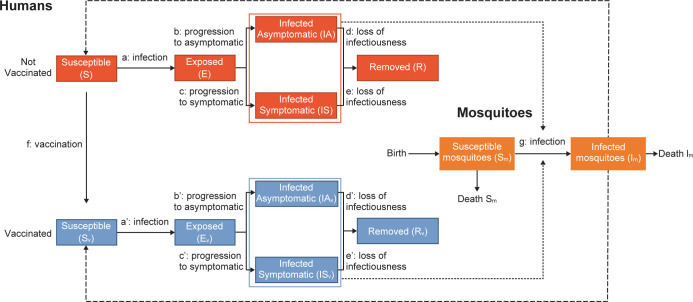
Schematic of the outbreak vector-borne transmission model. Note that the model is not serotype or serostatus specific. a: infection of humans depending on the number of infected vectors in the area and the neighboring eight areas. a’: same as a, accounting for vaccine efficacy against infection. b and b’: progression from incubation to asymptomatic state depending on vaccination status and efficacy against symptomatic outcome. birth: the number of new noninfected mosquitoes is equal to the number of dead mosquitoes (death I_m_ + death S_m_) in order to have a fixed number of vectors per area. c and c’: progression from incubation to symptomatic state depending on vaccination status and efficacy against symptomatic outcome. d, d’, e, and e’: loss of infectiousness of humans depending on vaccination status. E and E_v_: exposed humans nonvaccinated and vaccinated. f: vaccination. g: infection of mosquitoes depending on the number of infected humans in the area and their vaccination status. IAs and IAs_v_: infected asymptomatic humans nonvaccinated and vaccinated. I_m_: infected mosquitoes. IS and IS_v_: infected symptomatic humans nonvaccinated and vaccinated. R and R_v_: removed (immune or dead after infection) among humans nonvaccinated and vaccinated. S and S_v_: susceptible humans nonvaccinated and vaccinated. S_m_: susceptible mosquitoes.

The impact of vaccination on outbreak management was modeled using an age-structured, host-vector, spatial and stochastic transmission model, which included seasonality to reflect the variation in disease transmission across months. The model was based on the epidemiological data from the 2017 Sri Lankan dengue outbreak [1], and it simulated the transmission of dengue to both humans and mosquitoes in parallel using an interdependent infection process (i.e., infected humans infect susceptible mosquitoes and vice versa), with mosquitoes as the main driver of infection (Fig 2). In the population of infected humans, it was assumed that a proportion would develop clinical symptoms (see below) and that a proportion of these would require hospitalization. The rest were assumed to be asymptomatic infections. The model equations are shown in S1 Text.

The model was restricted to the district of Colombo (population of approximately 2.3 million [12]) because the highest number and greatest density of dengue cases were reported in this area during the 2017 outbreak [1] and it is where some phase 3 trial sites were located. The district was divided into 10,913 areas corresponding to squares of 250 m × 250 m. The spatial distribution of the population within each square was based on data from the Global Human Settlement Population Grid dataset [13], and demographics were obtained from the World Population Prospects dataset [14]. In the model, the inhabitants in each square were categorized into three age groups (infants [0–1 year old], children [2–16 years old], adults [17–99 years old]), and the population of mosquitoes was estimated in proportion to the number of inhabitants. Based on a flight radius of 200 m for *Ae*. *aegypti* mosquitoes [15], each area was considered directly linked to neighboring areas within this radius (i.e., eight neighboring squares), and it was assumed that mosquitoes from these areas could be infectious to these inhabitants.

The model was calibrated against overall incidence data collected by the Ministry of Health on the epidemiology of dengue in Sri Lanka in 2017 (S1 Fig) [1]. Time-dependent seasonal factors and probabilities of infection (from vectors and humans) were inferred using an optimization algorithm (subplex) implemented in the NLopt library, using maximum likelihood methodology (Table 1) [16]. An expansion factor of 10 was also applied to the calibrated incidence data to account for assumed underreporting of dengue cases [17]; this was considered a conservative approach compared with the expansion factor of 30 that was estimated in a previous analysis of dengue infection in children in Colombo [17]. We assumed that 80% of the population would be susceptible to infection by DENV-2, the dominant strain in the outbreak. Changes to the proportion of the susceptible population had an impact on the estimated transmission parameters but no impact on the estimated incidence hence little impact on our results (See model equations in S1 Text). The model assumed all individuals aged 4–60 years (1.85 million individuals), out of the population of approximately 2.3 million, were eligible for vaccination, regardless of dengue serostatus. Based on the parameters used in previous analyses [18], we have assumed that 30% of infected humans develop clinical symptoms, and the remaining 70% are asymptomatic. Because the contribution of asymptomatic infections to herd immunity and community infection are difficult to estimate, we have assumed that infected symptomatic individuals are twice as likely to transmit the virus than asymptomatic individuals [18].

**Table 1:** Key epidemiological model parameters.

Parameter	Value	Range for sensitivity analyses	Reference
VE against VCD caused by DENV-2 (first dose to 1 year post second dose)	95%	50%–95%	Biswal et al., 2020 [8]
VE against hospitalized VCD caused by DENV-2 (first dose to 1 year post second dose)	97%	50%–97%	NCT02747927
VE against asymptomatic cases	47%	25%–47%	Coudeville et al., 2016 [19]
Proportion of asymptomatic versus symptomatic cases	70%	–	Flasche et al., 2016 [18]
Duration from first dose to VE (S2 Fig)	14 days	14–30 days	NCT02747927
Outbreak incidence^a^	Calibrated	–	Tissera et al., 2020 [1]
Expansion factor	10	–	Tam et al., 2013 [17]
Probability of hospitalization (symptomatic cases)	70%	20%–80%	NCT02747927
Duration from outbreak start to beginning vaccination	30 days	15–180 days	Assumption
Target vaccination coverage	65%	10%–80%	Our World in Data, 2022 [20]
Duration of vaccination outbreak campaign	90 days	–	Assumption
Probability of infection from humans to mosquitoes	0.6736	–	Estimated during calibration^b^
Probability of infection from mosquitoes to humans	0.4645	–	Estimated during calibration^b^
Duration of infectiousness of mosquito^c^	1.31 days	–	Estimated during calibration^b^
Probability that a mosquito will move to a neighboring cell	0.6873	–	Estimated during calibration^b^
Duration of infectiousness for humans to mosquitoes^c^	7.6 days	–	Estimated during calibration^b^
Seasonality factor^d^	0.7017	–	Estimated during calibration^b^

^a^Observed overall incidence was used for the calibration and then adjusted by age.

^b^Parameters were estimated during calibration to reflect the 2017 epidemic curve.

^c^Comparable with Codeco et al. [21].

^d^Factor calculated to calibrate to 2017 epidemic curve.

DENV-2, dengue serotype 2; VCD, virologically confirmed dengue; VE, vaccine efficacy.

Because DENV-2 was the dominant strain during the 2017 outbreak, the VE assumptions used in the model were based on estimates against DENV-2 from part 1 of the clinical trial (95% against VCD [8] and 97% against hospitalized VCD cases in the overall population). VE against asymptomatic cases was assumed to be 50% of the VE against VCD (i.e., 47%), as used previously in the evaluation of the potential impact of another licensed dengue vaccine, CYD-TDV [19]. Efficacy estimates were applied across age groups. The probability of hospitalization was assumed at 70% based on the high rate observed among VCD cases in the placebo group in Sri Lanka during the phase 3 efficacy trial [2]. Sensitivity analyses using lower hospitalization rates of 50% and 20% were also performed.

### Scenarios

The reference scenario assumed the epidemiological situation in 2017 and no vaccination against dengue. We compared this with a vaccination baseline scenario that would achieve a coverage rate of 65% of the 4- to 60-year-old target population over a 90-day vaccination outbreak response campaign, with day 1 of the modeling corresponding to the day the 2017 outbreak was officially declared. This translates to a vaccination rate of 13,369 new persons dosed per day, assuming initiation of vaccination 30 days after the start of the outbreak. The vaccine was assumed to be effective 14 days after administration of the first dose based on the noted time of separation of the placebo and TAK-003 curves at the trial level in the overall population across all serotypes combined (S2 Fig).

Because the vaccination scenario was based on a number of variable epidemiological parameters (Table 1), we performed sensitivity analyses to assess the robustness of our results across uncertainties in VE, vaccination campaign start date, vaccination campaign intensity (i.e., number of new person doses administered per day), and probability of hospitalization. As part of this sensitivity analysis, we assessed “extreme high” (i.e., start vaccination on day 15, 80% coverage rate, baseline VE assumptions) and “extreme low” (i.e., start vaccination on day 180, 10% coverage rate, VE of 50% against VCD, 50% against hospitalization, and 25% against asymptomatic cases) scenarios.

In total, 100 individual simulation runs were performed. Data are presented as median values across runs, with corresponding 95% CIs. The model was developed using R.4.2.1 and C++ with a Shiny package interface, mainly using Rcpp 1.0.9, Rcpp Armadillo 0.11.2.3.1, and RcppGSL 0.3.11. Clinical data were analyzed using SAS version 9.3.

## Results

Of the 20,099 participants in the trial, 2,100 were included in the safety set in Sri Lanka, with 703 allocated to placebo and 1,397 to TAK-003. At enrollment, 38.3% (802/2,095) in the safety set (i.e., those who received at least one dose of TAK-003 or placebo) were seronegative to all four dengue serotypes as per a validated microneutralization assay [22], and nearly all (95.6%) had been vaccinated previously against Japanese encephalitis.

As of the clinical trial data extraction date of November 16, 2018, the incidence of VCD in the Sri Lankan placebo group from first dose of vaccination (between November 2016 and March 2017) until the end of part 1 (approximately 15 months later) was 9.3/100 person-years (p-y). When stratified by age, the incidence of VCD was 4.4/100 p-y among 4- to 5-year-old participants, 9.8/100 p-y among 6- to 11-year-old participants, and 10.0/100 p-y among 12- to 16-year-old participants. Most VCD cases in the placebo group were DENV-2 (76/81 cases). Sixty-nine percent of VCD cases were hospitalized in the placebo group, 94.6% of which were owing to DENV-2.

**Fig 3:**
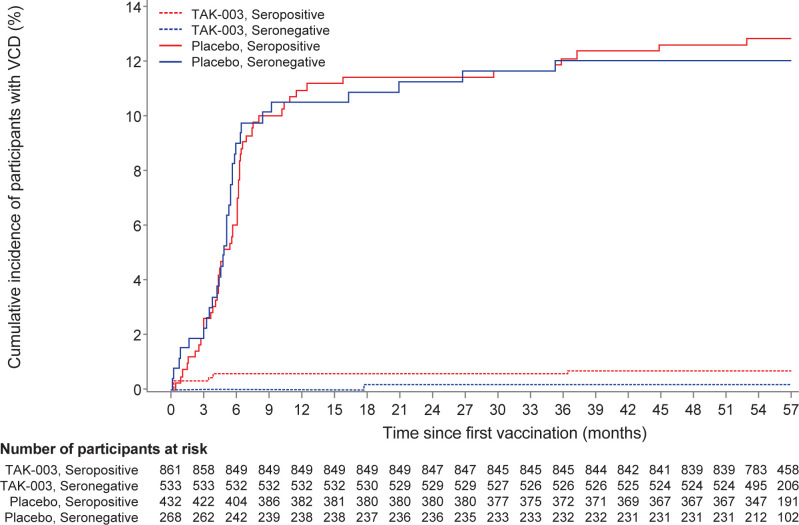
Cumulative incidence of VCD caused by dengue serotype 2 from first vaccine dose until 4.5 years after the second dose in participants in Sri Lanka by baseline serostatus (safety set).

In participants at sites in Sri Lanka, the VE of TAK-003 from first vaccination until the end of part 1 was 94.7% (95% CI 89.5%–97.4%; 81/700 cases for placebo, 9/1,394 for TAK-003) against VCD and 95.7% (95% CI 89.3%–98.3%; 56/700 for placebo, 5/1,394 for TAK-003) against hospitalized VCD. VE was similar in baseline seronegative and seropositive subpopulations (Table 2). When stratified by age, the incidence of VCD was 0.6/100 p-y for both 6- to 11-year-old participants and 12- to 16-year-old participants. The cumulative VCD incidence caused by DENV-2 showed an early and wide separation of TAK-003 compared with placebo, irrespective of baseline serostatus (Fig 3).

**Table 2:** VE against VCD and hospitalized VCD from first vaccination until the end of part 1 in participants at trial sites in Sri Lanka (safety set).

	No. of cases in TAK-003 group	TAK-003 cases per 100 p-y	No. of cases in placebo group	Placebo cases per 100 p-y	VE % (95% CI)
VCD					
Overall	9 (n = 1394^a^)	0.5	81 (n = 700^a^)	10.1	94.7 (89.5–97.4)
Serostatus					
Seropositive	8 (n = 861^a^)	0.8	52 (n = 432^a^)	10.5	92.7 (84.7–96.5)
Seronegative	1 (n = 533^a^)	0.2	29 (n = 268^a^)	9.4	98.4 (87.9–99.8)
Hospitalized VCD	5 (n = 1394^a^)	0.3	56 (n = 700^a^)	6.8	95.7 (89.3–98.3)

^a^Number of participants evaluated.

Baseline seronegative was defined as seronegative to all serotypes; baseline seropositive was defined as having a reciprocal neutralizing antibody titer of ≥ 10 to one or more serotypes.

p-y, person-years; VCD, virologically confirmed dengue; VE, vaccine efficacy.

As of the data extraction date of March 21, 2022 (approximately 4.5 years after the second dose [end of part 3]), 103 cases of VCD have been recorded at Sri Lankan trial sites in the placebo group from the first dose onward. Of these, 78.6% (81/103) occurred during part 1 of the trial (coinciding with the dengue outbreak). From first vaccination until the end of part 3 (approximately 4.5 years after the second dose), the VE of TAK-003 in Sri Lanka was 84.4% (95% CI 77.0%–89.4%; 101/700 cases for placebo, 34/1,394 for TAK-003) for preventing VCD cases by any dengue serotype. The VE of TAK-003 for preventing hospitalization owing to VCD was 87.9% (95% CI 79.6%–92.8%; 70/700 cases for placebo, 18/1,394 for TAK-003).

To date, the vaccine has been well tolerated across participants in all trial sites, including those in Sri Lanka (S3 Table for safety data from participants in Sri Lanka) [2,7–9].

### Modeling

**Fig 4:**
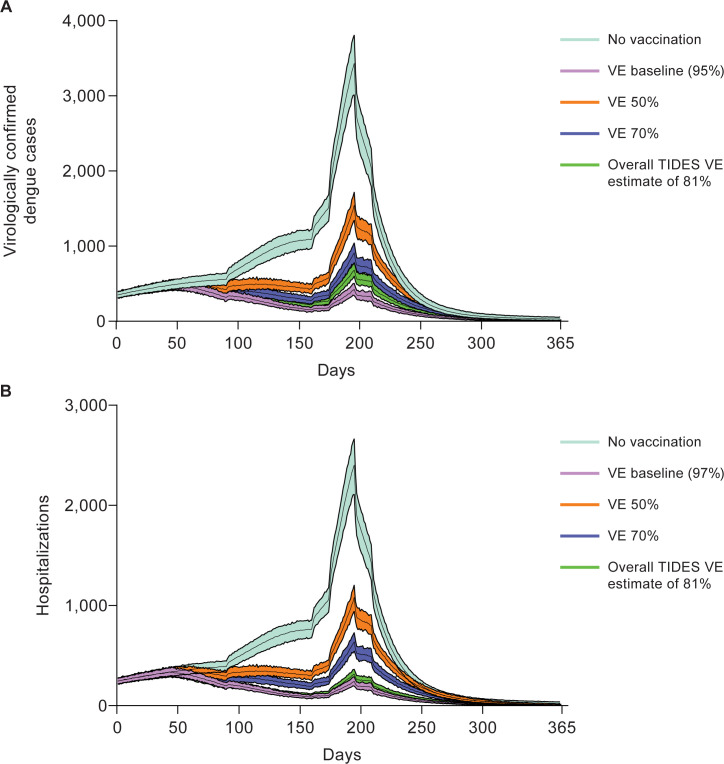
Sensitivity analysis of VE and epidemic curves of (A) VCD and (B) hospitalized VCD.

Because the majority of dengue cases occurred in people aged 4–60 years, vaccinating these age groups was expected to directly impact the shape of the epidemic curve. Based on the initial scenario assumptions (“VE baseline”; Table 1) and part 1 trial data, flattening of the case curve was observed from approximately 70 days post vaccination onward, avoiding the outbreak (i.e., exponential rise). In contrast, without vaccination, the exponential increase in cases began at approximately 160 days for both VCD cases (Fig 4A) (see S3 Fig for cumulative VCD) and hospitalizations (Fig 4B). Use of the overall phase 3 trial VE estimate (81% from first vaccination until the end of part 1 in the safety analysis set across all serotypes and all countries) produced an outbreak response curve similar to that generated for the VE estimate based on DENV-2 alone (Fig 4). Overall, substantial reductions in VCD cases could be achieved with 65% target coverage rate (baseline scenario), and the shape of this outbreak curve was similar to the curve generated when using a scenario with a coverage rate of 80% (S4 Fig). However, a 10% target coverage rate did not substantially impact the shape or peak of the outbreak curve compared with no vaccination.

**Fig 5:**
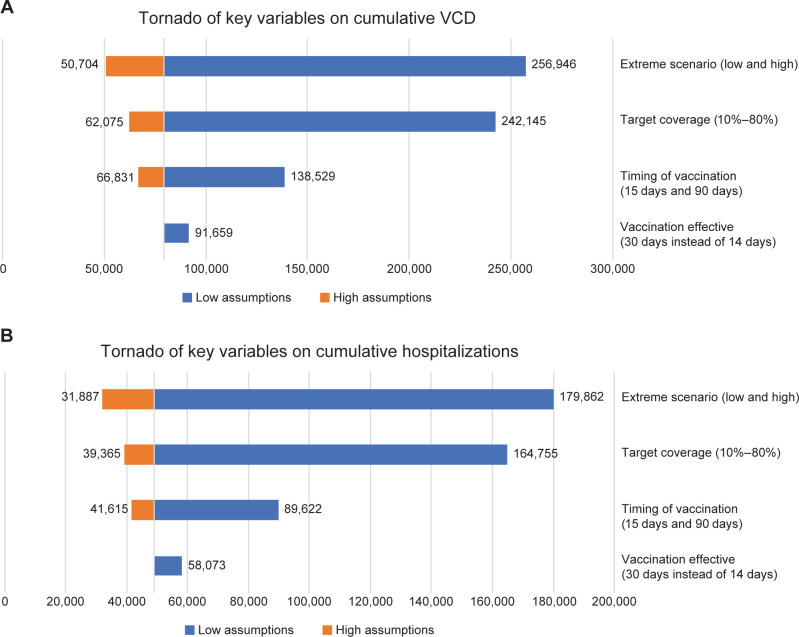
Tornado plots of the effect of key variables on the cumulative number of (A) VCD cases caused by DENV-2 and (B) hospitalized VCD cases caused by DENV-2. Extreme low scenario: start vaccination on day 180, 10% coverage, vaccine efficacy 50%, 50% hospitalization, and 25% asymptomatic. Extreme high scenario: start vaccination on day 15, 80% coverage, and vaccine efficacy baseline.

Sensitivity analyses were also performed for different vaccination timings, intensities, and efficacy scenarios. All scenarios reduced the number of VCD and hospitalized VCD cases compared with no vaccination (S4 Table). The assumption of 80% of the eligible population being susceptible to DENV-2 infection was based on the observed incidence from the 2017 epidemic. However, varying this percentage did not result in any changes in the model outcomes. The baseline vaccination scenario resulted in a 69.1% reduction in VCD cases and a 72.7% reduction in VCD hospitalizations compared with the no vaccination scenario. When the VE was instead assumed to be 50% and 70%, the number of VCD case rates prevented was reduced to 41.1% and 56.0%, respectively (S4 Table). Shorter intervals to commencing vaccination, lower intervals between vaccination and effectiveness, and vaccine coverage also independently reduced the overall number of VCD and hospitalized VCD cases (Fig 5). Overall, when all the factors were combined, the extreme low scenario (start of vaccination 180 days after the outbreak, a coverage of 10%, and 50% VE against both VCD and hospitalized cases and 25% against asymptomatic cases) only marginally controlled the outbreak, whereas the extreme high scenario resulted in an 80.3% and 82.3% reduction in cases of VCD and hospitalized VCD, respectively, compared with no vaccination (S4 Table).

## Discussion

The main objective of conducting the TAK-003 pivotal efficacy trial in eight countries was to assess the vaccine in different epidemiological settings and to increase the chance of capturing enough dengue cases of different serotypes to assess its efficacy. As we had anticipated, disease burden and serotype distribution patterns varied between countries. In Sri Lanka, the trial population included approximately 62% who were pre-exposed to dengue, the vast majority had a prior Japanese encephalitis vaccination, and most of the cases had accrued in the early part of the trial during the 2017 outbreak.

In a previous exploratory evaluation, we reported that two doses of TAK-003 administered 3 months apart was efficacious in all participating countries and that the magnitude of VE estimates was largely dependent on the prevalent serotype in those countries because of varying performance of TAK-003 against individual serotypes [7–9]. Overall, TAK-003 has shown to be well tolerated and efficacious in the Sri Lankan population for ≥ 4.5 years.

In areas with dengue outbreaks, the principal burden is created by the enormous number of hospitalizations and, therefore, the resources to care for these patients [23,24]. For instance, as noted in the trial, dengue case management in the regions of Sri Lanka included in the study involved high rates of hospitalization as well as close monitoring through frequent radiological examination and laboratory investigations [2]. This suggests substantial health care utilization. Flattening the outbreak curve would ensure that health care facilities are not overwhelmed and have the capacities to manage illnesses other than dengue.

Modeling of the trial data to an extended geographic area showed a substantial reduction in the number of cases and a flattening of outbreak curves. The 2017 outbreak in Sri Lanka was primarily caused by DENV-2 and, therefore, our base model considered the efficacy estimate of TAK-003 against this serotype. Although this is the best-case scenario from the point of view of vaccine performance, DENV-2 is also responsible for the highest number of mono-infection outbreaks, followed by dengue virus serotype 1 (DENV-1) [25]. Coincidentally, this is the order of TAK-003 performance by serotype, i.e., highest efficacy against DENV-2 followed by DENV-1 [2,7–9]. Both serotypes are also the most commonly reported in outbreaks involving multiple serotypes [25]. TAK-003 efficacy varies by serotype, with a higher efficacy against DENV-2 compared with other serotypes [6,9].

In the pivotal trial TIDES, serotype specific VE point estimates were 79.4% against DENV-1, 95.2% against DENV-2, 60.3% against DENV-3 and 58.5% against DENV-4 until the first 12 months after the vaccination. The corresponding estimates until 54-months were 52.3%, 82.8%, 42.6% and 48.3%, respectively [26]. These resulted in overall efficacy of 80.9% and 61.2% in these two timeframes, respectively. Notably, we observed generally higher efficacy against hospitalized cases that persisted over time, which is important from a public health or societal burden perspective [6,7]. Further exploratory analysis indicated that TAK-003 was efficacious against all 4 DENV serotypes in baseline seropositive participants and against DENV-1 and -2 in baseline seronegative participants. Data also suggested lack of protection against DENV-3 in seronegative participants whilst the performance against DENV-4 could not be assessed in this population owing to the small number of cases [6]. Therefore, the overall impact of vaccination in a given outbreak setting will be determined by the infecting serotype and the seroprevalence in that population as well as the above vaccine performance attributes. Interestingly, modeling VE levels of 50% still resulted in flattening of the outbreak curves, suggesting that even modestly efficacious vaccines could play an important role in outbreak control.

Besides the vaccine’s attributes, the modeling data indicated that the speed of vaccination and coverage were other key drivers of vaccination impact on an outbreak. Starting vaccination earlier in the outbreak resulted in substantial reductions in estimated numbers of cases, whereas a response that did not start until 180 days into the outbreak resulted in a very limited reduction in VCD cases (6% compared with no vaccination). Similarly, a vaccine coverage of 10% would have very little impact on case numbers, whereas 80% coverage would reduce case numbers by approximately 75%. A significant effect of vaccination could still be realized with a modestly efficacious vaccine when vaccination is implemented rapidly with an optimum level of coverage. It is likely that routine vaccination would mitigate outbreaks and aid in reducing the number of additional doses, which would be needed as part of an outbreak response. The recent COVID-19 experience has highlighted the complexities of vaccine procurement and vaccination implementation, and has indicated that a target coverage of approximately 65% is a reasonable estimate for Sri Lanka [20]. Therefore, apart from close monitoring of epidemiology and vaccine performance, public health officials need to take several other factors into account in order to consider TAK-003 vaccination as an option in dengue outbreak control. Ready availability of adequate doses and proactive planning are therefore crucial.

There are several limitations to our analysis and modeling approach. The trial was not designed to assess results at the country level, and the results are exploratory in nature. Therefore, results must be considered in the context of the overall trial results, local epidemiology, and the observations derived from an exploratory analysis. In addition, it does not take into consideration differences in VE by dengue serotype, although various VE levels were taken into consideration for the sensitivity analysis to account for varying serotype specific efficacy and mixed serotype outbreaks. As discussed above, we expect that the overall impact in an outbreak setting will be dependent on the serotype mix among other factors and should be taken into consideration. The VE for the clinical trial was calculated for participants aged 4–16 years, whereas the model included adults. To keep the modeling methodology straightforward, many complexities were not considered such as mixed serotype outbreaks; age-specific incidence, hospitalization rates, or efficacy estimates; potential impact of vaccination on transmission; impact of symptomatic versus asymptomatic cases on transmission; various geographic and epidemiological settings; case management practices; primary, secondary, or postsecondary infections; cross-protection; and the nature of outbreaks once routine dengue vaccination is the norm. However, we believe the supportive sensitivity analyses address this to a large extent, and inclusion of these other parameters would likely not change the conclusions drawn from the model.

In conclusion, TAK-003 was well tolerated and efficacious in Sri Lankan children and adolescents aged 4–16 years. A supportive modeling analysis suggests TAK-003 has the potential of meaningful utility in dengue outbreaks in endemic areas. Vaccine performance, speed of vaccination campaign initiation, and vaccine coverage are important factors of the likely impact.

## Supporting information

S1 Text Model equations and additional safety and efficacy information. (DOCX)

S1 Table Overview of TAK-003 VE by serotype against VCD and hospitalized VCD from first vaccination until the end of part 1 and the end of part 3 (safety set). (DOCX)

S2 Table Institutional review boards (IRBs) and independent ethics committees (IECs). (DOCX)

S3 Table Overview of solicited and unsolicited AEs after any vaccination in the safety set immunogenicity subset, and serious AEs in the safety set in participants in Sri Lanka. (DOCX)

S4 Table Sensitivity analysis of the absolute and marginal (percentage difference vs. no vaccination) numbers of VCD and hospitalized VCD cases for vaccination start, vaccination intensity, and vaccine efficacy. (DOCX)

S1 Fig Calibration of the model against published data from the 2017 dengue outbreak in Sri Lanka. (DOCX)

S2 Fig Cumulative incidence of VCD from first vaccination until second vaccination (per protocol set). (DOCX)

S3 Fig Cumulative analysis of (A) VCD and (B) hospitalized VCD. (DOCX)

S4 Fig Sensitivity analysis of vaccine coverage and epidemic curves of VCD in different scenarios. (DOCX)
