# Supplementary material for: Role of the dengue vaccine TAK-003 in an outbreak response: Modeling the Sri Lanka experience
Source: PLoS Negl Trop Dis. 2024 Aug 22;18(8):e0012376. doi: 10.1371/journal.pntd.0012376 (PMC11419351; doi:10.1371/journal.pntd.0012376)
Supplement: S1 Text — (DOCX) [file pntd.0012376.s001.docx]

**Model equations**

The model equations for the vector infection process, human infection process and the likelihood of infections are:

***Vector infection proces****s*

where $IV_{i}\left( t \right)$ is the number of infected vectors in area $i$ at time $t$, $SV_{i}\left( t \right)$ is the number of susceptible vectors in area $i$ at time $t$, $\beta_{m}$ is the probability of infection of a vector by a human, $IH_{i}\left( t \right)$ and $vIH_{i}\left( t \right)$ are the number of non-vaccinated and vaccinated infected humans in area $i$ at time $t$, $w_{asym}$ is the reduction of transmission factor for asymptomatic infected, $VE_{trans}$ is the vaccine effectiveness against transmission, $AH_{i}\left( t \right)$ and $vAH_{i}\left( t \right)$ are the number of non-vaccinated and vaccinated asymptomatic infected humans in area $i$ at time $t$, $N_{i}$ is the number of humans in area $i$, $IV_{i}\left( t \right)$ is the number of infected vectors in area $i$ at time $t$, $\mu_{v}$ is the probability of loss of infectious status or death of the vector. The function $seasonality\left( t \right)$ gives the seasonal factor at time $t$ and is computed as the product between a “seasonality force factor” (seas_fact) and the normalized weekly number of cases observed by the surveillance system in order to obtain the right incidence trend. Here, we assume that the number of vectors (susceptible and infected) remains constant in time, an assumption which is compensated by the seasonality factor used in the human infection process.

***Human infection process***

where $flow_{X-Y,i}\left( t \right)$ describes the flow of human from status $X$ to status $Y$ in area $i$ between time $t$ and $t+1$, $SH_{i}\left( t \right)$ is the number of susceptible humans in area $i$ at time $t$, the operator $neighbors\left( i \right)$ give the references to the 8 areas neighboring $i$, $NV_{j}$ is the total number of vectors in $j$, $\beta_{h}$ is the probability of infection of a human by a vector, $VE_{inf}$ is the vaccine effectiveness against infection, $p_{symp}$ is the probability of symptomatic infection, $RVE_{symp}$ is the relative vaccine effectiveness against clinical symptoms, $\gamma$ is the probability of loss of infection among humans. The function $seasonality\left( t \right)$ gives the seasonal factor at time $t$ and is computed as the product between a “seasonality force factor” (seas_fact) and the normalized weekly number of cases observed by the surveillance system in order to obtain the right incidence trend.

***Likelihood***

The daily number of human infected is assumed to follow a $Poisson$ distribution. Under this assumption, the likelihood function can be written as:

$$L=\sum_{t} dpois\left( Cases\left( t \right),IH\left( t \right) \right)$$

where $dpois\left( x,\lambda\right)$ gives the log density for $x$ of the $Poisson$ distribution of parameter $\lambda$, $Cases\left( t \right)$ is the observed number of dengue cases at time $t$.

**Safety**

***Methodology***

Safety measures were assessed after each vaccine dose. Solicited local adverse events (AEs) were evaluated for participants of any age in the safety set immunogenicity subset and included injection site pain, injection site erythema and injection site swelling within 7 days of each vaccine dose. Solicited systemic AEs included fever (>38°C) evaluated for participants of any age; drowsiness, loss of appetite and irritability/fussiness evaluated for participants aged <6 years; and headache, myalgia, malaise and asthenia for participants aged ≥6 years within 14 days of each vaccine dose. Unsolicited AEs up to 28 days were evaluated in the safety set immunogenicity subset.

With the exception of solicited local AEs that were automatically considered related to vaccine administration, all other AEs were evaluated by the investigators for relatedness to vaccine administration. SAEs, AEs leading to vaccine dose discontinuation or discontinuation from the trial, and AEs leading to death were evaluated in the safety set throughout the trial. Results are presented descriptively.

***Results***

Among the Sri Lankan population in trial DEN-301, more solicited local and systemic adverse events were reported by TAK-003 recipients than placebo recipients; however, the proportion of vaccine-related solicited systemic AEs was similar in both groups (S2 Table). The frequency of unsolicited AEs was similar in both groups with none being vaccine-related or leading to discontinuation from vaccine dose or the trial. More SAEs were reported among placebo than TAK-003 recipients at the end of Part 1 (12 months after the second vaccine dose) and there were no deaths due to AEs in either group. These findings are similar to those reported in the global population at year 1 and also at later timepoints in parts 2 and 3 of the trial [1-4].

**Efficacy**

***Methodology***

Full details of evaluation of efficacy in the trial DEN-301 have been published previously [2]. In brief, the primary end point was the vaccine efficacy of two doses of TAK-003 for the prevention of virologically confirmed dengue (VCD) induced by any dengue virus serotype from 30 days after the second injection until the end of part 1 of the trial. Secondary vaccine efficacy end points included efficacy against individual dengue virus serotypes, efficacy according to baseline serostatus, and efficacy for the prevention of hospitalized VCD and the prevention of severe dengue until the end of part 2 of the trial.

Analysis of the primary end point was performed with the per-protocol population, which included all participants who did not have any major protocol violations. Vaccine efficacy is defined as 1 minus the hazard ratio (vaccine vs. placebo). Hazard ratios and corresponding 95% confidence intervals were estimated with a Cox proportional-hazards model that included trial group as a factor, with adjustment for age and stratification according to region. Additional analyses were performed with the per protocol population, safety population, full analysis population, or safety and immunogenicity subpopulations.

**References**

1. Biswal S, Borja-Tabora C, Martinez Vargas L, Velasquez H, Theresa Alera M, Sierra V, et al. Efficacy of a tetravalent dengue vaccine in healthy children aged 4-16 years: a randomised, placebo-controlled, phase 3 trial. Lancet. 2020;395(10234):1423-33. doi: <https://doi.org/10.1016/S0140-6736(20)30414-1>. PMID: 32197105.

2. Biswal S, Reynales H, Saez-Llorens X, Lopez P, Borja-Tabora C, Kosalaraksa P, et al. Efficacy of a tetravalent dengue vaccine in healthy children and adolescents. N Engl J Med. 2019;381(21):2009-19. doi: <https://doi.org/10.1056/NEJMoa1903869>. PMID: 31693803.

3. López-Medina E, Biswal S, Saez-Llorens X, Borja-Tabora C, Bravo L, Sirivichayakul C, et al. Efficacy of a dengue vaccine candidate (TAK-003) in healthy children and adolescents 2 years after vaccination. J Infect Dis. 2022;225(9):1521-32. doi: <https://doi.org/10.1093/infdis/jiaa761>. PMID: 33319249.

4. Rivera L, Biswal S, Sáez-Llorens X, Reynales H, López-Medina E, Borja-Tabora C, et al. Three-year efficacy and safety of Takeda's dengue vaccine candidate (TAK-003). Clin Infect Dis. 2022;75(1):107-17. doi: <https://doi.org/10.1093/cid/ciab864>. PMID: 34606595.
