# Supplementary material for: Role of the dengue vaccine TAK-003 in an outbreak response: Modeling the Sri Lanka experience
Source: PLoS Negl Trop Dis. 2024 Aug 22;18(8):e0012376. doi: 10.1371/journal.pntd.0012376 (PMC11419351; doi:10.1371/journal.pntd.0012376)
Supplement: S1 Table — (DOCX) [file pntd.0012376.s002.docx]

**S1 Table. Overview of TAK-003 VE by serotype against VCD and hospitalized VCD from first vaccination until the end of part 1 and the end of part 3 (safety set)**

|  | **VE (95% CI)** | |
| --- | --- | --- |
|  | **VCD** | **Hospitalized VCD** |
| **End of part 1** |  |  |
| DENV-1 | 79.4 (64.9–87.9) | 75.1 (-36.2–95.4) |
| DENV-2 | 95.2 (90.4–97.6) | 97.3 (91.5–99.2) |
| DENV-3 | 60.3 (41.2–73.2) | 66.8 (-17.5–90.6) |
| DENV-4 | 58.5 (-36.1–87.3) | 100.0 (NE) |
| **End of part 3** |  |  |
| DENV-1 | 52.3 (42.6–60.3) | 71.2 (51.2–82.9) |
| DENV-2 | 82.8 (77.3–86.9) | 97.0 (92.6–98.8) |
| DENV-3 | 42.6 (26.2–55.3) | 47.6 (0.2–72.5) |
| DENV-4 | 48.3 (8.4–70.8) | 100.0 (NE) |

DENV, dengue virus serotype; NE, not evaluable; VCD, virologically confirmed dengue; VE, vaccine efficacy
