# Supplementary material for: Role of the dengue vaccine TAK-003 in an outbreak response: Modeling the Sri Lanka experience
Source: PLoS Negl Trop Dis. 2024 Aug 22;18(8):e0012376. doi: 10.1371/journal.pntd.0012376 (PMC11419351; doi:10.1371/journal.pntd.0012376)
Supplement: S2 Table — (DOCX) [file pntd.0012376.s003.docx]

**S2 Table. Institutional review boards (IRBs) and independent ethics committees (IECs)**

| **Central IRB/IECs** | Comissao Nacional de Etica em Pesquisa (CONEP) -Unidade II do Ministerio da Saude SEPN 510-Norte -Bloco A-1o Subsolo- Edificio Brazil |
| --- | --- |
|  | Comité de Ética en la Investigación CAIMED Carrera 42 No. 17-50 Santa Fe de Bogotá D.C. Cundinamarca 111611 Colombia |
|  | Ethics Review Committee Faculty of Medical Sciences, University of Sri Jayawardenepura, Gangodawila, Nugegoda, 10250 Sri Lanka |
| **Local IRB/IECs** | Comitê de Ética em Pesquisa do Hospital Universitário Cassiano Antônio de Moraes – CEP/HUCAM Avenida Marechal Campos, 1355-Santos Dumond Vitória, Espírito Santo 29040-001 Brazil |
|  | Comité de Ética em Pesquisa do Hospital Santo Antônio / Obras Sociais Irmã Dulce Av. Bonfim, 161 Largo de Roma Salvador BA 40415-000 Brazil |
|  | Comitê de Ética em Pesquisa em Seres Humanos da Universidade Federal de Mato Grosso do Sul-UFMS Cidade Universitária -Caixa Postal 549 Pró-Reitoria de Pesquisa e PósGraduação-PROPP Campo Grande MS 79070-900 Brazil |
|  | Comitê de Ética em Pesquisa da Liga Norte Riograndense Contra o Câncer Rua Dr Mário Negócio, 2267 Quintas Natal-RN 59040-000 Brazil |
|  | Corporación Científica Pediátrica-Comité de Ética en Investigación Biomédica Calle 5 B5 N0 37 Bis-28 Cali-Valle del Cauca 760021 Colombia |
|  | Comité de Bioética de Investigación del Hospital Maternidad Nuestra Señora de la Altagracia Calle Pedro Henríquez Ureña #49 Santo Domingo República Dominicana |
|  | Comité de Ética para Investigaciones Biomédicas (CEIB) Universidad Nacional Autónoma de Nicaragua UNAN, Campo Médico León Nicaragua |
|  | Comité de Bioética en Investigación del Hospital del Niño Panamá, Ave. Balboa, Calle 34 0816-00383 Panamá República De Panamá |
|  | University of the Philippines Manila Research Ethics Board 2/F Paz Mendoza, 547 Pedro Gil St., Ermita Manila, 1000 Philippines |
|  | Research Institute for Tropical Medicine Institutional Review Board Filinvest Corporate City, Alabang Muntinlupa, 1781 Philippines |
|  | Independent Ethics Committee De La Salle Health Science Institute Ground Floor De La Salle Angelo King Medical Research Center Congressional Avenue Dasmarinas, Cavite, 4114 Philippines |
|  | Walter Reed Army Institute of Research Institutional Review Board, 503 Robert Grant Avenue, Silver Spring, MD, 20910-7500 USA |
|  | Chong Hua Institutional Review Board Chong Hua Hospital Don Mariano Cui Street, Fuente Osmeña Cebu City, 6000 Philippines |
|  | Ethical Review Committee for Research in Human Subjects Ministry of Public Health The Office of the Secretary, Department of Medical Services 3^rd^ Floor Of the building No.2, Ministry of Public Health Muang, Nonthaburi, 11000 Thailand |
|  | Ethics Committee of the Faculty of Tropical Medicine, Mahidol University 4^th^ floor, the 60^th^ Ann. Of King´s Accession to the Throne Building Faculty of Tropical Medicine, Mahidol University Bangkok, 10400 Thailand |
|  | Ethics Committee of Buddhasothorn hospital 174 Marupong Road, Muang District Chachoengsao, 24000 Thailand |
|  | Office of the Khon Kaen Ethics Committee in Human Research Academic and Research Laboratory Building (Wechwichakarn), 3^rd^ Floor Room 5317 Faculty of Medicine Khon Kaen University Khon Kaen, 40002 Thailand |
|  | The Institutional Review Board, Royal Thai Army Medical Department Pharmongkutklao College of Medicine, 317 Rajavithi Road, Rajathevee Bangkok, 10400 Thailand |
