# Supplementary material for: Role of the dengue vaccine TAK-003 in an outbreak response: Modeling the Sri Lanka experience
Source: PLoS Negl Trop Dis. 2024 Aug 22;18(8):e0012376. doi: 10.1371/journal.pntd.0012376 (PMC11419351; doi:10.1371/journal.pntd.0012376)
Supplement: S3 Table — (DOCX) [file pntd.0012376.s004.docx]

**S3 Table. Overview of solicited and unsolicited AEs after any vaccination in the safety set immunogenicity subset, and serious AEs in the safety set in participants in Sri Lanka**

|  | **TAK-003, n/N (%)** | **Placebo, n/N (%)** |
| --- | --- | --- |
| Local^a^ solicited AEs | 88/266 (33.1) | 31/139 (22.3) |
| Systemic^b^ solicited AEs | 95/266 (35.7) | 38/139 (27.3) |
| Related | 14/266 (5.3) | 8/139 (5.8) |
| Unsolicited AEs^c^ | 50/266 (18.8) | 23/142 (16.2) |
| Related | 0 (0) | 0 (0) |
| Leading to discontinuation | 0 (0) | 0 (0) |
| Serious AEs^d^ | 87/1,394 (6.2) | 104/700 (14.9) |
| Deaths^e^ | 0/1,394 (0.0) | 0/700 (0.0) |

Data are presented for the number of participants reporting one or more events.

^a^Occuring within 7 days after vaccination.

^b^Occuring within 14 days after vaccination.

^c^Up to 28 days after any vaccination.

^d^Up to end of part 1 (i.e., 1 year after second dose). Two cases of dengue were reported as being related to the study vaccine by the investigator (data remain blinded because the study is ongoing).
^e^Up to end of part 1 (i.e., 1 year after second dose).

AE, adverse event.
