# Supplementary material for: Role of the dengue vaccine TAK-003 in an outbreak response: Modeling the Sri Lanka experience
Source: PLoS Negl Trop Dis. 2024 Aug 22;18(8):e0012376. doi: 10.1371/journal.pntd.0012376 (PMC11419351; doi:10.1371/journal.pntd.0012376)
Supplement: S4 Table — (DOCX) [file pntd.0012376.s005.docx]

**S4 Table. Sensitivity analysis of the absolute and marginal (percentage difference vs. no vaccination) numbers of VCD and hospitalized VCD cases for vaccination start, vaccination intensity, and vaccine efficacy**

| **Scenario** | **VCD cases**  **(median, 95% CI)** | **Hospitalized VCD cases**  **(median, 95% CI)** | **Median percentage reduction versus no vaccination** | |
| --- | --- | --- | --- | --- |
|  |  |  | **VCD, %** | **Hospitalized VCD, %** |
| No vaccination | 256,976  (223,020–292,507) | 179,883  (156,114–204,754) | – | – |
| Baseline scenario^a^ | 79,335  (65,844–92,288) | 49,123  (41,537–57,813) | 69.1 | 72.7 |
| Variation in VE |  |  |  |  |
| VE: 50% VCD and hospitalization, 25% asymptomatic | 151,254  (130,764–173,348) | 105,877  (91,535–121,344) | 41.1 | 41.1 |
| VE: 70% VCD and hospitalization, 35% asymptomatic | 113,162  (96,069–131,722) | 79,213  (67,249–92,206) | 56.0 | 56.0 |
| Variation in vaccination start date |  |  |  |  |
| Vaccination starts on day 15 | 66,831  (55,612–79,353) | 41,615  (34,774–49,219) | 74.0 | 76.9 |
| Vaccination starts on day 90 | 138,529  (120,056–157,906) | 89,622  (77,738–102,055) | 46.1 | 50.2 |
| Vaccination starts on day 180 | 242,404  (212,593–270,110) | 167,617  (147,115–186,589) | 5.7 | 6.8 |
| Variation in vaccination effectiveness interval |  |  |  |  |
| Vaccination effective from day 30 | 91,659  (77,776–106,031) | 58,073  (49,441–67,013) | 64.3 | 67.7 |
| Target vaccination coverage |  |  |  |  |
| Target coverage 10% | 242,145  (210,838–275,270) | 164,755  (143,454–187,295) | 5.8 | 8.4 |
| Target coverage 80% | 62,075  (52,138–72,807) | 39,364  (33,213–45,996) | 75.8 | 78.1 |
| Hospitalization rate |  |  |  |  |
| 20% (vs. no vaccination with 20% hospitalization) | 79,335  (65,844–92,288) | 14,331  (45,047–58,340) | 69.1 | 72.1 |
| 50% (vs. no vaccination with 50% hospitalization) | 79,335  (65,844–92,288) | 35,589  (29,896–41,429) | 69.1 | 72.3 |
| Combined extreme scenarios |  |  |  |  |
| Extreme high (start vaccination on day 15, 80% coverage target, baseline VE) | 50,704  (41,811–60,609) | 31,887  (26,466–37,898) | 80.3 | 82.3 |
| Extreme low (start vaccination on day 180, 10% target coverage, VE 50% for VCD, 50% for hospitalized VCD, 25% for asymptomatic) | 256,946  (222,253–292,304) | 179,862  (155,577–204,313) | 0.0 | 0.0 |

^a^Vaccination starts at 30 days, 65% target coverage, vaccine is effective at 14 days, 70% hospitalization rate. VE: 95% for VCD, 97% for hospitalized VCD, and 47% for asymptomatic.
VCD, virologically confirmed dengue; VE, vaccine efficacy.
