# Supplementary figures and images for: Role of the dengue vaccine TAK-003 in an outbreak response: Modeling the Sri Lanka experience
Source: PLoS Negl Trop Dis. 2024 Aug 22;18(8):e0012376. doi: 10.1371/journal.pntd.0012376 (PMC11419351; doi:10.1371/journal.pntd.0012376)

**S1 Fig. Calibration of the model against published data from the 2017 dengue outbreak in Sri Lanka [1]**


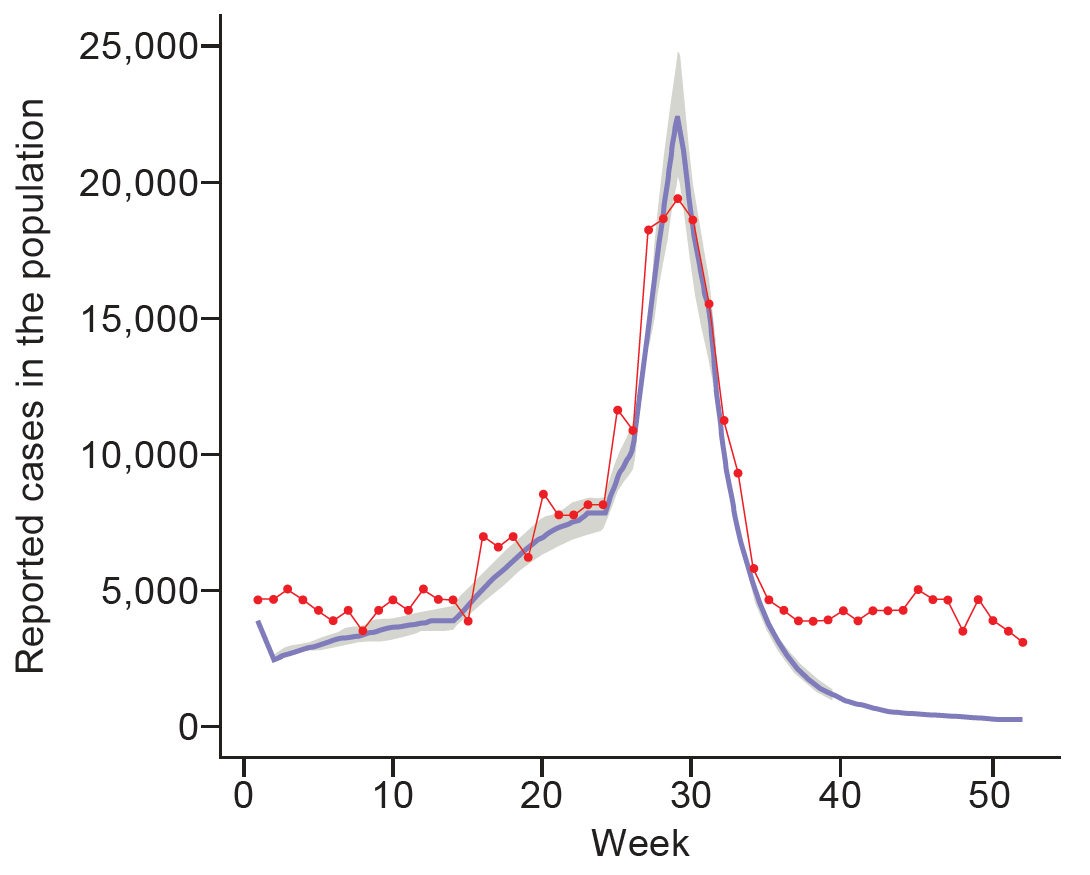

Supplement: S1 Fig — (DOCX) [file pntd.0012376.s006.docx]

**S3 Fig. Cumulative analysis of (A) VCD and (B) hospitalized VCD**


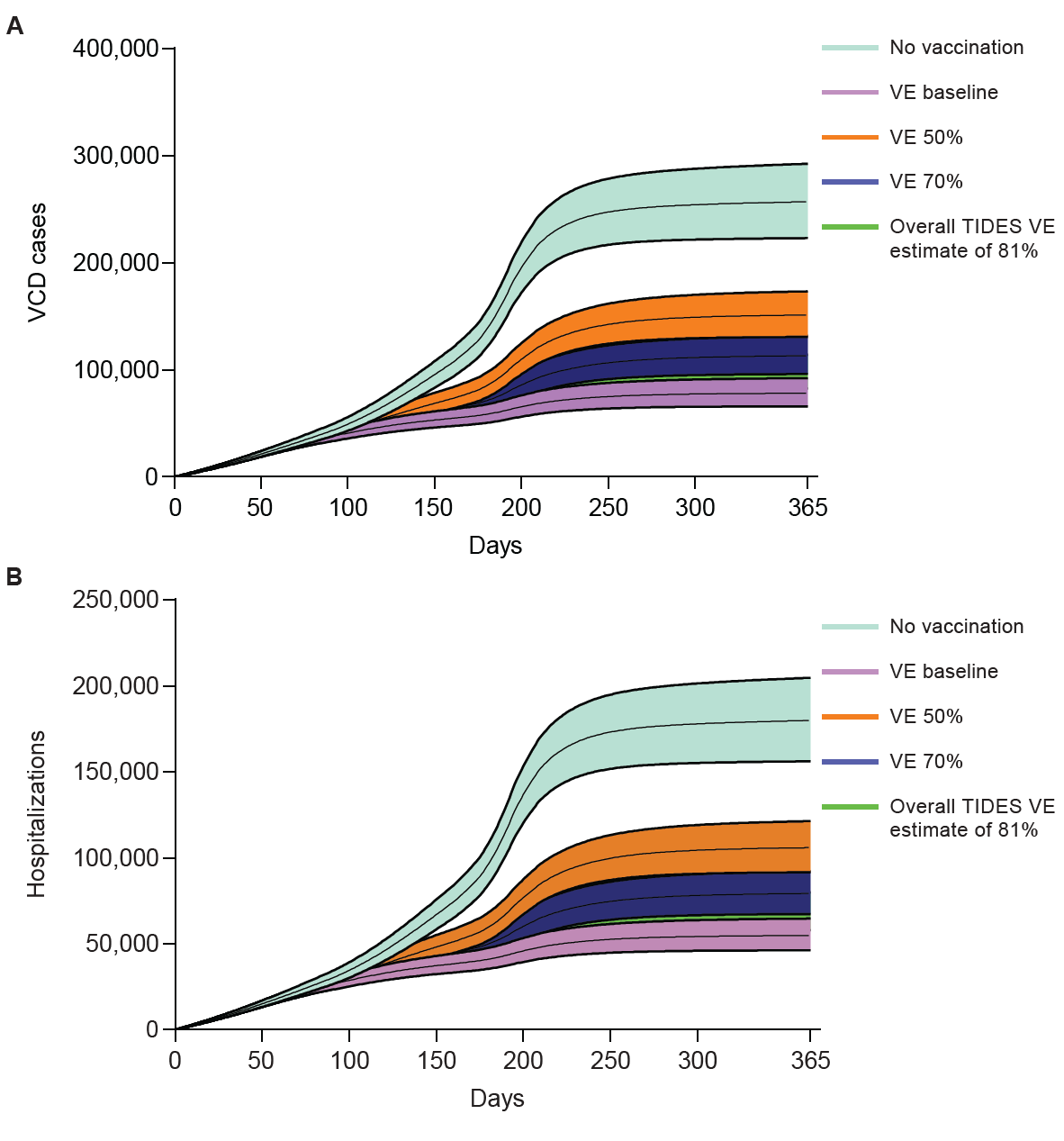

Supplement: S3 Fig — (DOCX) [file pntd.0012376.s008.docx]
