# Supplementary material for: Role of the dengue vaccine TAK-003 in an outbreak response: Modeling the Sri Lanka experience
Source: PLoS Negl Trop Dis. 2024 Aug 22;18(8):e0012376. doi: 10.1371/journal.pntd.0012376 (PMC11419351; doi:10.1371/journal.pntd.0012376)

**S4 Fig. Sensitivity analysis of vaccine coverage and epidemic curves of VCD in different scenarios**


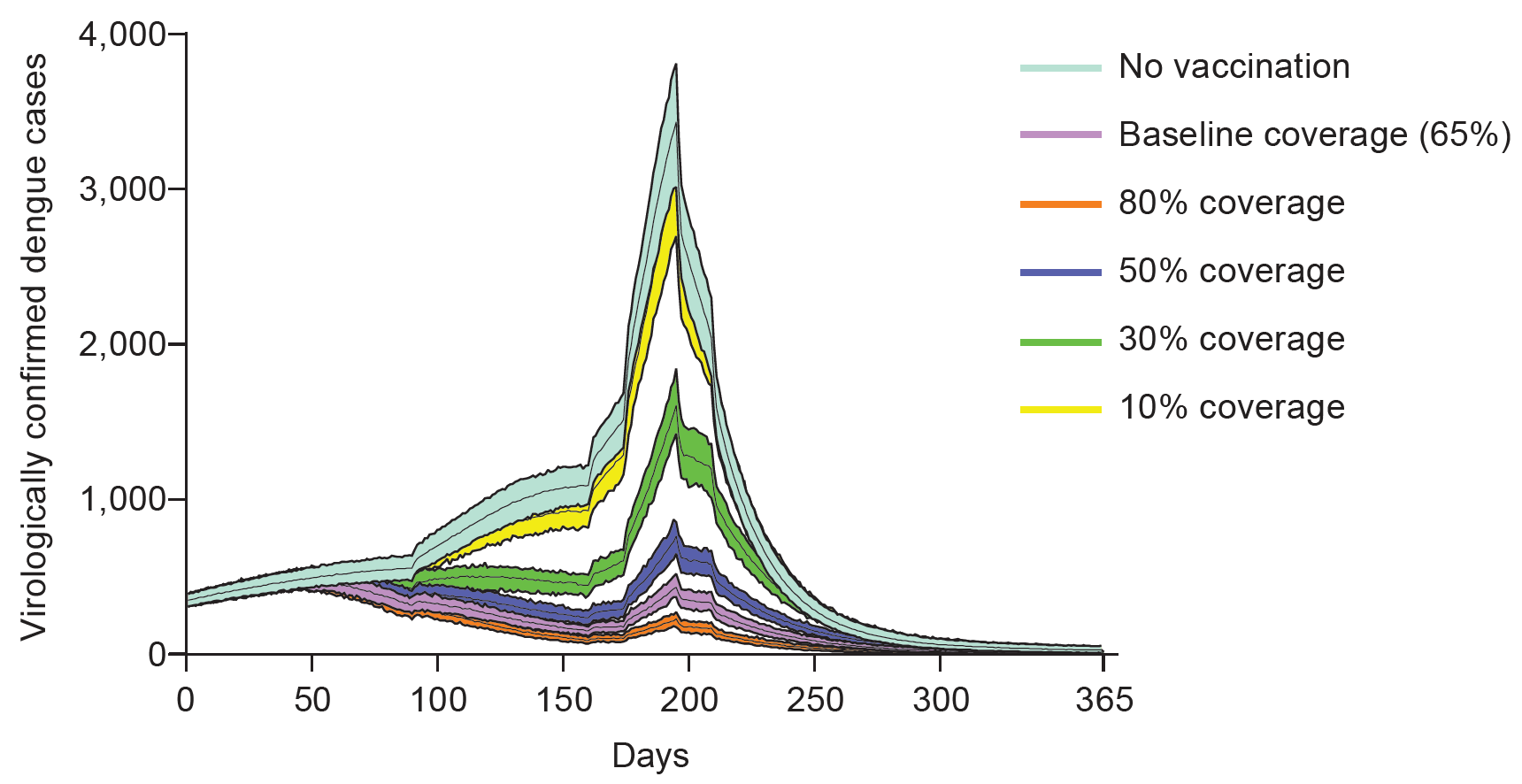

Supplement: S4 Fig — (DOCX) [file pntd.0012376.s009.docx]
